# The antihyperglycemic potential of pyrazolobenzothiazine 1, 1-dioxide novel derivative in mice using integrated molecular pharmacological approach

**DOI:** 10.1038/s41598-023-49932-2

**Published:** 2024-04-02

**Authors:** Saman Taj, Usman Ali Ashfaq, Matloob Ahmad, Hasnat Noor, Ayesha Ikram, Rashid Ahmed, Muhammad Tariq, Muhammad Shareef Masoud, Anwarul Hasan

**Affiliations:** 1 Department of Bioinformatics and Biotechnology, Government College University Faisalabad, Faisalabad, 38000 Pakistan; 2 Department of Chemistry, Government College University Faisalabad, Faisalabad, 38000 Pakistan; 3 Department of Biotechnology, Faculty of Natural and Applied Sciences, Mirpur University of Science and Technology, New Mirpur City, 10250 Pakistan; 4 Department of Mechanical and Industrial Engineering, Qatar University, 2713, Doha, Qatar; 5 Biomedical Research Center (BRC), Qatar University, PO Box 2713, Doha, Qatar

**Keywords:** Biochemistry, Computational biology and bioinformatics, Drug discovery, Diseases, Materials science

## Abstract

Diabetes Mellitus is a metabolic disease characterized by elevated blood sugar levels caused by inadequate insulin production, which subsequently leads to hyperglycemia. This study was aimed to investigate the antidiabetic potential of pyrazolobenzothiazine derivatives in silico, in vitro*,* and in vivo. Molecular docking of pyrazolobenzothiazine derivatives was performed against α-glucosidase and α-amylase and compounds were selected based on docking score, bonding interactions and low root mean square deviation (RMSD). Enzyme inhibition assay against α-glucosidase and α-amylase was performed in vitro using *p*-nitrophenyl-α-D-glucopyranoside (PNPG) and starch substrate. Synthetic compound pyrazolobenzothiazine (S1) exhibited minimal conformational changes during the 100 ns MD simulation run. S1 also revealed effective IC50 values for α-glucosidase (3.91 µM) and α-amylase (8.89 µM) and an enzyme kinetic study showed low ki (− 0.186 µM, − 1.267 µM) and ki′ (− 0.691 µM, − 1.78 µM) values with the competitive type of inhibition for both enzymes α-glucosidase and α-amylase, respectively. Moreover, studies were conducted to check the effect of the synthetic compound in a mouse model. A low necrosis rate was observed in the liver, kidney, and pancreas through histology analysis performed on mice. Compound S1 also exhibited a good biochemical profile with lower sugar level (110–115 mg/dL), increased insulin level (25–30 μM/L), and low level of cholesterol (85 mg/dL) and creatinine (0.6 mg/dL) in blood. The treated mice group also exhibited a low % of glycated haemoglobin (3%). This study concludes that S1 is a new antidiabetic-agent that helps lower blood glucose levels and minimizes the complications associated with type-II diabetes.

## Introduction

Diabetes is a rapidly spreading metabolic endocrine disease across the globe. Diabetes mellitus (DM) is the precursor of other metabolic disorders categorized by high blood sugar levels due to fault in insulin secretion or function. Postprandial hyperglycemia refers to elevated glucose levels after the meal: linked with microvascular and macrovascular diseases, mainly neuropathy, retinopathy, nephropathy, and cardiovascular disorders. These conditions pose significant challenges for individuals, as they can be difficult to cope with due to the potential for complications and the impact on overall health and well-being^1^. The International Diabetes Federation (IDF) reported that 463 million people are affected by type II diabetes globally. Pakistan ranks fourth, with 19 million diabetic patients, a number projected to rise to 26 million by 2030^2^. A recent collaborative study by the University of Manchester in the United Kingdom and the Pakistan Endocrine Society has revealed alarming statistics regarding diabetes in Pakistan. Among individuals aged 20–70 years, the study found that 8.5 percent of the population is currently living with diabetes. Even more concerning is the prevalence rate of type II diabetes, which stands at an alarming 16.98 percent. Within this group of individuals with type II diabetes, 10.91 percent are classified as pre-diabetic cases^3,4^. These findings underscore the urgent need for effective strategies and interventions to address the growing diabetes epidemic in Pakistan.

The correlation between chemistry and computational biology is profound and multifaceted. Computational biology is a field that uses mathematical and computational techniques to analyze and model biological data and processes. Chemistry, on the other hand, is the science that deals with the composition, structure, properties, and changes of matter. Computational biology relies heavily on chemistry when it comes to drug discovery. Computational methods can be used to simulate and analyze the interactions between drug molecules and biological targets (such as proteins)^5^. Understanding these interactions at the molecular level is crucial for designing and optimizing drugs for various diseases. Computational biology often involves molecular modelling, which requires a deep understanding of the chemical properties of biomolecules. Computational chemists and biologists collaborate to develop accurate models of biological molecules like proteins and nucleic acids, which are essential for studying their functions and interactions. Many bioinformatics tools and software used in computational biology are based on algorithms and techniques from computational chemistry^6^. These tools help researchers analyze biological data, predict protein structures, and simulate biological processes. Computational methods, such as molecular dynamics simulations and quantum chemistry, play a crucial role in determining the three-dimensional structures of biomolecules. This information is essential for understanding how proteins fold, interact with other molecules, and carry out their biological functions^7^.

In the small intestinal villi, α-glucosidase, a membrane-bound enzyme, is responsible for breaking down complex sugar oligosaccharides into simpler sugars. These monosaccharides are subsequently absorbed into the bloodstream to meet the body's energy requirements. Saliva contains α-amylase, another enzyme involved in the breakdown of oligosaccharides^8^. Blocking the catalytic activity of both enzymes α-glucosidase and α-amylase can lead to a delay in carbohydrate metabolism and absorption, resulting in undigested carbohydrates being eliminated from the body, thus lowering blood glucose levels^9^. There are several medications available as α-glucosidase and α-amylase blockers such as acarbose and miglitol. These drugs inhibit the enzyme’s catalytic activity, ultimately preventing the absorption of sugars from the intestines^10–12^. Consequently, these inhibitors can effectively mitigate the expression of the disease and acarbose and miglitol have proven to be valuable antidiabetic agents. However, acarbose and voglibose are poorly absorbed by gut, have low bioavailability and are excreted in the stool. On the other hand, miglitol is well absorbed by the gut and is excreted through the renal route. Miglitol and voglibose do not produce metabolites primarily due to their minimal metabolic transformation. In contrast, acarbose undergoes metabolic processes in the colon, leading to the production of various metabolites as a result of enzymatic activity^13^. These inhibitors can induce side effects such as diarrhea and flatulence^14–17^.

Shifting the focus to α-amylase, this calcium metalloenzyme is responsible for cleaving internal α-1,4-glycosidic bonds in starch, producing glucose and maltose^13^. Carbohydrates, as primary energy source for the body, play an indispensable role in daily nutrition. The intricate components of dietary carbohydrates necessitate conversion into simpler sugars through the collaborative action of α-amylase and α-glucosidase^15^. This transformation enables the absorption of glucose from the intestine into the bloodstream. Inhibiting the actions of α-glucosidase and α-amylase offers a promising therapeutic approach for mitigating carbohydrate absorption and reducing blood glucose levels^6^. Amylase blockers hold therapeutic significance and are considered as potential medications for diabetes management^5,16^.

Keeping in view the imporatnce of amylase blockers for reducing risks of diabetes, we used a pyrazolobenzothiazine 5,5 dioxide derivative named “(E)-4-Hydroxy-N′-(1-phenylethylamine)-2H-benzo[e][1,2]thiazine-3-carbohydrazide1,1-dioxide as an antidiabetic agent. We evaluated its potential in silico by molecular docking studies, and we performed enzyme inhibition assay using p-nitrophenyl-α-D-glucopyranoside and starch substrate in vitro*.* For in vivo studies, we used mouse models as these animals share over 80 percent similar genetic components with humans^17,18^. In this study, the synthetic compound pyrazolobenzothiazine (S1) demonstrated potent inhibitory effects on α-glucosidase and α-amylase with low IC50 values, signifying its potential as an antidiabetic agent. Moreover, in a mouse model, S1 exhibited favorable outcomes, including reduced blood sugar levels, increased insulin levels, and minimal complications associated with type II diabetes, suggesting its promise as a novel therapeutic option for managing the condition.

## Materials and methods

### Molecular docking

#### Ligand preparation

**Figure 1:**
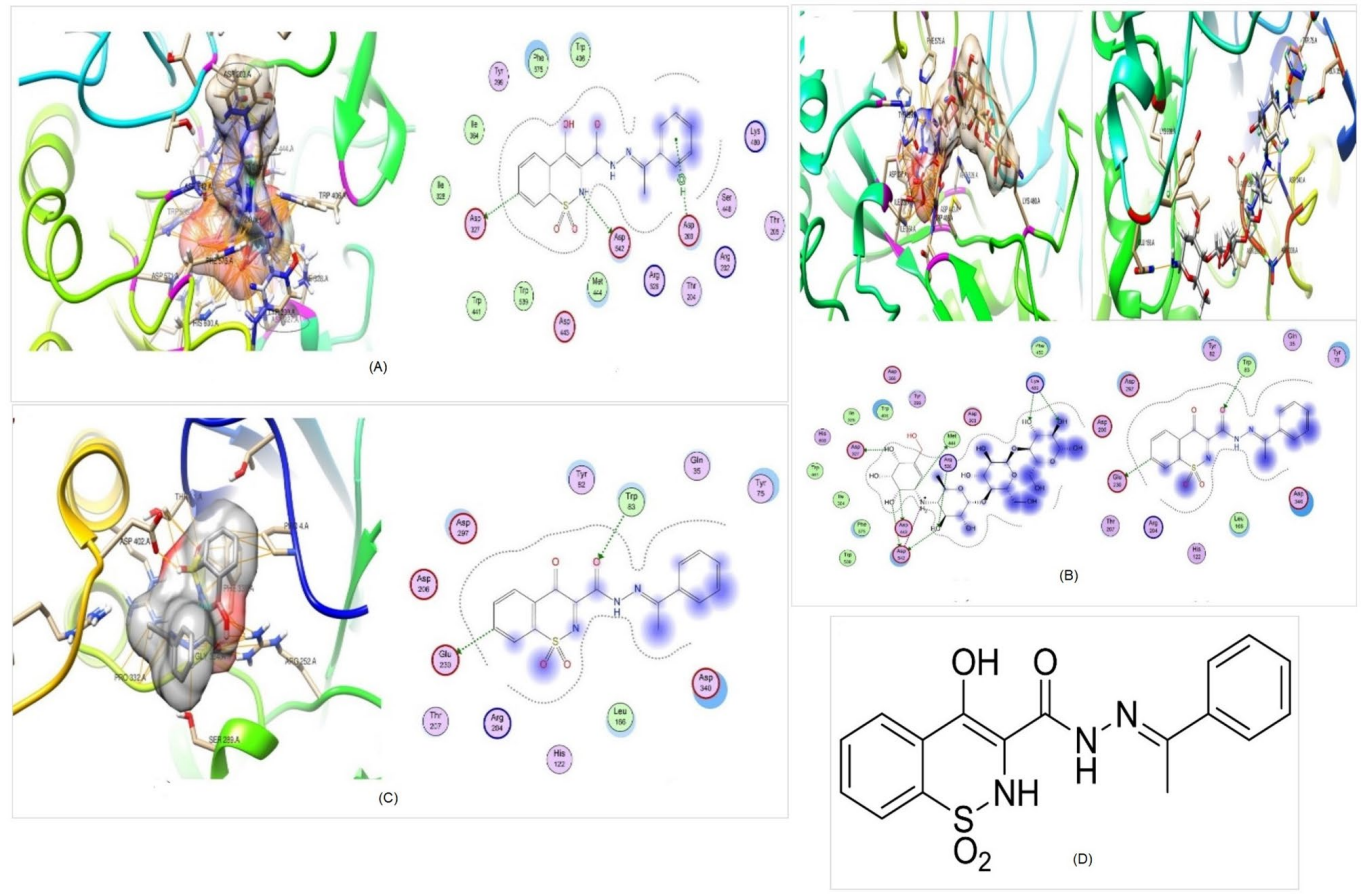
Molecular docking studies of synthetic compounds with protein α-glucosidase and α–amylase (PDB ID: 2QMJ and &TAA respectively) performed through MOE software on default parameters. (**A**), Compound S1 3D map and 2D interaction against α-glucosidase, the red ring indicates acidic amino acids, the blue ring indicates basic residues, purple and green circle indicates polar and hydrophobic residues, respectively. (**B**), Compound S1, 3D map and 2D interaction against α-amylase, the red ring indicates acidic amino acids, the blue ring indicates basic residues, and the purple and green circle indicates polar and hydrophobic residues, respectively. (**C**), 3D map and interaction of control with α-glucosidase and with α-amylase, the red ring indicates acidic amino acids, the blue ring indicates basic residues, and purple and green circle indicates polar and hydrophobic residues respectively. (**D**), 3D structure of the compound.

ChemDraw Ultra 12.0, a molecule editor, was used to draw the 2D structure of synthetic compounds (Fig. 1D) in ‘.sdf’ format. Optimization of ligands was done through MOE (2014.0901) by adding partial charges on ligands using protonate 3D to ionize certain residues of ligands under the experiment. Energy minimization was done via the MMFF94X force field. MMFF94 performs well at improving calculations, bond lengths, and points and incorporates electrostatic and hydrogen-holding effects. The optimized ligands were then added to the MOE ligand database for further docking study.

#### Receptor preparation

A Protein Data Bank (PDB) was utilized to retrieve the 3D structure of both proteins. PDB ID: 2QMJ used for the α-glucosidase enzyme and α-amylase enzyme PDB ID: 7TAA was used. The 3D structure of the protein was optimized by removing already bounded ligands and solvent molecules of protein. A site finder was used to find the docking site of the protein molecule, and dummies were generated at the binding pocket (size 64 residues) containing Asp203, Asp542, Asp327, Arg526, and His600 residues for α-glucosidase. We selected the same site for binding on which the standard drug acarbose was bound. A binding pocket containing Trp83, Asp340, Arg344, Arg Asp204, Glu230, and Lys209 residues was selected for α-amylase and the size of the binding pocket is 107. Both proteins were ionized separately by adding partial charges through protonate 3D (Electrostatics: GB/VI, dielectric: 2, van der Waals: 800R3) and energy minimization was performed through the MMFF94X force field.

Following parameters (Rescoring 1: London dG, Refinement: Force field, Rescoring 2: London dG, Placement: Triangle matcher, retain: 10) were set to run docking of α-glucosidase and α-amylase with ligand molecule at a specific site. In the wake of docking, the best and top conformity were selected based on interacting residues, S-score, and RMSD value^18,19^.

### ADMET analysis (Drug likeness analysis)

Drug-likeness and ADMET analysis predict the clinical preliminaries necessary to discover a drug that improves the chance of a compound being used as an oral drug. It tends to be characterized as the quality of the sub-atomic physicochemical properties that are normal for synthetic substances known as drugs. The drug-likeness study was done through Lipinski’s rule of five, Egen rule, Veber’s rule and GSK 4/400 rule for compound S1 with the best docking score and interactions at the binding sites. Molinspiration server (https://www.molinspiration.com/) was used to determine physicochemical properties (molecular weight, log P-value, no. hydrogen bond acceptor, no. of hydrogen bond donor, no of rotatable bonds, etc.) to determine the ADMET (Absorption, Distribution, Metabolism, Excretion, and the Toxicity) parameters of compound S1 the admetSAR database was used.

### Molecular dynamics simulation

Complex S1 was taken for molecular dynamics simulation examination. Desmond v3.6 module (2019) from the Schrodinger suite was used to conduct molecular dynamics simulation for 1–100 ns. To understand the key physicochemical processes of condensed phase structure, their motion at the molecular and atomic levels was analyzed. Furthermore, it is compulsory to determine their interaction to analyze structural conformations between target and ligand. The system was set at 1 atm pressure and 300 K temperature to carry molecular dynamic simulation by applying an NPT ensemble. To mimic physiological conditions salt concentration of the system was set at 0.15M. OPLS_2005 force fields were utilized for the simulation study. RMSF and RMSD of protein and ligand molecules were examined to evaluate the simulation study.

### In vitro analysis

#### Sample preparation

Pyrazolobenzothiazine derivatives were synthesized according to our reported methodology^20^. For further in vitro analysis, compound S1 was prepared in Dimethyl sulfoxide (DMSO, Sigma–Aldrich) with a stock concentration of 10 mM and kept at room temperature. To get the maximum working concentration of the test compound, the stock was diluted into DMSO at 500 µM concentration.

#### α-glucosidase inhibition assay

To carry out this reaction, 10 μL (500 µM) of 10 mM sample solution (prepared in DMSO) was added to ELISA 96 microwell plate. Then, 40 μL of 0.5U/ml α glucosidase was mixed by adding 120 μL of 0.1M phosphate buffer with a *p*H value of 7.4. The solution was incubated at 37 °C for 5 min. The reaction proceeded further by adding 40 μL of 5 mM substrate solution (PNPG) and incubation was done for half an hour at 37 °C. After performing the incubation microplate reader was used to read the absorbance of the reaction mixture at 405 nm wavelength. Acarbose was used as the positive control. The inhibition percentage of the sample and positive control were calculated using the following formula.

$$\text{Inhibition } \% = \left[ {\frac{B - S}{B} } \right]*100$$

At this point, the B and S denote absorbance for the blank and samples. Blank contains only DMSO instead of sample.

#### α-amylase inhibition assay

ELISA plate was used for inhibition assay and it was loaded with 40 µL of the sample. Enzyme prepared in 0.02 M sodium phosphate buffer with pH 6.9 (40 µL) was further added to wells. The solution in wells was kept on incubation at 37 °C for 10 min. Moreover, 40 µL starch solution prepared in 1% DMSO was added to the solution and put on incubation for 10 min at 25 °C. At last, to stop the reaction, 100 µL of DNSA reagent was added to the solution. The mixture was put in a boiling water bath for 5 min and cooled down at room temperature after incubation. For dilution of the reaction mixture, 400 µL of distilled water was added to the solution, and the absorbance of the reaction mixture was measured at 540 nm wavelength. To calculate the percentage inhibition the following formula was used.

$$\text{Inhibition } \% = \left[ {\frac{B - S}{B}} \right]*100$$

At this point, the B and S denote absorbance for the blank and samples. Blank contains only DMSO instead of sample.

### Kinetics study (α-glucosidase)

The IC50 value of compound S1 was 3.91 for α-glucosidase and the Lineweaver–Burk plot was plotted for kinetic study. α-glucosidase enzyme concentration was constant while varying concentration (1 mM, 3 mM, 5 mM, 7 mM and 9 mM) of substrate [S] (p-nitrophenyl-α-D-glucoside) was used for reaction. In addition, varying concentrations (0.1 mM, 0.25 mM, 0.5 mM, 0.75 mM, 1 mM) sample/inhibitor [V] was used. The type of inhibition was determined by the Lineweaver–Burk plot, while the Michaelis Menten equation was used to find the value of the inhibition constant.

$$Vo = \frac{Vmax\left[ S \right]}{{Km + \left[ S \right]}}$$

$$\frac{1}{Vo} = \frac{Km + \left[ S \right]}{{Vmax\left[ S \right]}} = \frac{km}{{Vmax }}\frac{1}{S} + \frac{1}{Vmax}$$

The initial rate of reaction is indicated by V0, Michaelis Menten's constant signifies km, Km/Vmax represents to slope, and 1/Vmax denotes the y-intercept. The value of the inhibitory constant was calculated by plotting the secondary graph. Straight-line produced by slope versus [V] intersecting x-axis indicates ki value while ki′ was produced by intersecting line plotted between y-intercept and [V].

#### Kinetics study (α-amylase)

Compound S1 showing an IC50 value of 8.89 μM was further analyzed for the Lineweaver–Burk plot. Varying concentrations (0.25%, 0.5%, 1%, 1.5%, 2%) of substrate [S] (starch) were used with constant enzyme concentration. Same as substrate, varying concentration (1 mM, 3 mM, 5 mM, 7 mM, 9 mM) of the inhibitor [V] was used. Lineweaver–Burk plots were used to determine the type of enzyme inhibition and the Michaelis Menten equation was used to find the value of inhibition constant.

$$Vo = \frac{Vmax\left[ S \right]}{{Km + \left[ S \right]}}$$

$$\frac{1}{Vo} = \frac{Km + \left[ S \right]}{{Vmax\left[ S \right]}} = \frac{km}{{Vmax }}\frac{1}{S} + \frac{1}{Vmax}$$

The initial reaction rate is indicated by V0, Michaelis Menten's constant signifies km, Km/Vmax represents slope, and 1/Vmax denotes the y-intercept. The value of the inhibitory constant was calculated by plotting the secondary graph. Straight-line produced by slope versus [V] intersecting x-axis indicates ki value while ki′ was produced by intersecting line plotted between y-intercept and [V].

### In vivo study

#### Diabetes induction

All experimental protocols adhered to the guidelines outlined in the NIH Guide for Care and Use of Laboratory Animals and the ARRIVE guidelines for animal experimentation. The Animal Care and Use Committee (ACUC) of Government College University Faisalabad (GCUF-2021-09) provided approval for the study. For in vivo study, BALB/c mice (weight 26–33 g) were acquired from the Department of Pharmacy and kept in the optimized environment (12 h dark, 12 h light) with a regular pellet diet and normal tab water. Diabetes induction in mice was done through a standard protocol reported by^21^ with slight amendments. Alloxan monohydrate 150 mg/kg (following the body weight of mice) was liquified in normal saline and injected intraperitoneally in mice fasted for 16 h^22,23^. Furthermore, screening of mice was done based on their blood glucose levels and mice with blood glucose levels more than 1.26 g/L were considered diabetic, and therefore taken for further experiment/examination. The mice diagnosed with diabetes were classified into five groups and five mice in each group. The five groups were comprised of A (Non-diabetic/ Control group), B (not treated diabetic/ Diabetic control), C (Acarbose positive control), D (compound S1 with low dose 3.9 mg/kg), E (compound S1 with high dose 7.8 mg/kg). Two doses were selected because they indicate how the organism's response changes at different levels of exposure. This information is crucial for determining safe dosage levels for humans or animals.

#### Biochemical and histological examinations

For biochemical testing and histological examination, mice from each group were collected, and data were produced for different testing parameters, including fasting blood glucose, serum creatinine, serum insulin, HbA1c, triglyceride and cholesterol. A Countour TS glucometer was used to measure mice's fasting blood glucose levels. While Clinical Chemistry Reagents SRL (CCR) kit was used to measure serum creatinine and triglyceride levels. Moreover, the VITRO SCIENT kit was utilized to measure the serum insulin, cholesterol and HbA1c levels. For histological analysis, mice were sacrificed and their organs (liver, kidney and pancreas) were washed with cold saline and preserved in a 10% formalin solution. A compound microscope from the Department of Physiology, Government College University Faisalabad, was used for tissue investigation under a controlled and organized environment. Pictures of tissue samples were taken through OPTIKA software.

### Statistical analysis

Comparison of the control group with the treated group is assessed through one-way ANOVA, and a t-test was executed for within-group comparison through GraphPad prism v 8.0.2.

### Ethical approval

The study was conducted according to the guidelines of the Declaration of Helsinki, and approved by the Institutional Review Board (or Ethics Committee) of Government College University Faisalabad.

## Results

### Molecular docking

Molecular docking was done to find the conformations of ligands with α-glucosidase (PDB: 2QMJ) and α-amylase (PDB: 7TAA). Different parameters including RMSD (root mean square deviation) value, least binding affinity, and interacting residues were used to assess the best complex. The RMSD value (root mean square value) was used to calculate the distance among the atoms of constituents of the complex. The most stable binding pose would have the lowest RMSD value. Binding affinity is the strength of the binding interaction between a single biomolecule (e.g., protein or DNA) to its ligand/binding partner (e.g., drug or inhibitor). Binding affinity must be low in negative value to represent a stable and robust interaction. Residues involved in the binding also play an important role in determining the best pose. Attachment of ligands with the active site residues can help to filter out potential ligands.

Pyrazolobenzothiazine derivatives were docked against α-glucosidase and α-amylase and compound S1 was selected that showed the lowest RMSD 1.189 as compared to acarbose (2.142) for α-glucosidase. Against α-amylase S1 also shows low RMSD (2.508) as compared to acarbose control (2.812). S1 also shows the highest binding affinity (in minus) − 10.618 kcal/mol, − 8.208 kcal**/**mol for α-glucosidase and α-amylase compared to control − 11.236 kcal**/**mol and − 12.089 kcal/mol, respectively (Table 1). S1 shows interaction with α-glucosidase by 3 residues Asp542, Asp203, and Asp327, which have acidic nature all residues show H-bond interactions (Fig. 1A).

**Table 1:** Docking score, RMSD and interacting residues.

Compound	Docking score (kcal/mol)		RMSD		Interaction residues	
	α-glucosidase	α-amylase	α-glucosidase	α-amylase	α-glucosidase	α-amylase
S1	− 10.618	− 8.208	1.189	2.508	Asp542 Asp327 Asp203	Trp83 Glu230
Acarbose	− 11.236	− 12.089	2.142	2.812	Asp542 Asp327 Asp327 Arg526	Asn298 Ays209 Asp340

While in control acarbose, two residues Asp542 and Asp327 show an acidic nature and 1 residue Arg526 has a basic nature. Compared with acarbose S1 the lower RMSD, higher docking score, and more no. of residues correlated with the high inhibitory potential of S1 against α-glucosidase. While α-amylase S1 shows 2 H-bond interactions within the binding pocket through Trp83 and Glu230 in which one is hydrophobic and the other is acidic (Fig. 1B). Acarbose with α-amylase exhibit 3 binding interaction Asn298, Ays209, and Asp340 (Fig. 4). 2D and 3D interaction of the ligand with receptor also shows the best pose for binding with α-glucosidase (Fig. 1A) and α-Amylase (Fig. 1B) compared to standard acarbose (Fig. 1C).

### ADMET analysis

A molinspiration server was utilized to predict the ADMET properties of compound S1 (Table 2). Drug safety profiling and oral bioavailability tests were passed by compound S1. For further authentication, the drug-like properties of the compound were analyzed using admetSAR. The result suggests that compound S1 can be used as a drug against the α-glucosidase enzyme because S1 is a non-carcinogen and doesn’t cross the blood–brain barrier (Table 2). S1 also checked for a different drug-like property and results show that S1 passed the Lipinski rule of five as the drug has less molecular weight and logp value than standard drug acarbose (Table 3). S1 also fulfils the parameters of the Veber rule (No. of rotatable bonds < 10, TPSA value ≤ 140 Å) and Egan rule (TPSA and log p values ≤ 132 and 6 respectively). After all these analysis results show that S1 has drug-like properties and can be used as an oral drug as it passes the oral bioavailability standards.

**Table 2:** ADMET profiling of compound S1.

Compound		ADMET analysis															
				P-gp			Cyp450 substrate			CYP450 Inhibitor							
	BBB	HIA	Solubility	Substrate	Inhibition	ROCT	2C9	2D6	3A4	1A2	2C9	2D6	2C19	3A4	CYP IP	AMES	Carcinogens
S1	−	+	− 3.25	+	−	−	+	−	−	−	−	−	−	−	Low	−	−

*BBB* blood–brain barrier; *HIA* human intestinal absorption; *CYP450* cytochrome P450; *CYP IP* CYP inhibitory promiscuity; *ROCT* renal organic cation transportation; + present; − not present.

**Table 3:** Oral bioavailability and drug-like properties of compound S1.

Compound	Lipinski RO5	Egen rule	Veber rule	ADMET	GSK4/400 rule
S1	✓	✓	✓	✓	✓

✓ Compounds fulfilled the criteria, ✕ couldn’t pass the criteria.

### Molecular dynamics simulation

#### RMSD analysis

**Figure 2:**
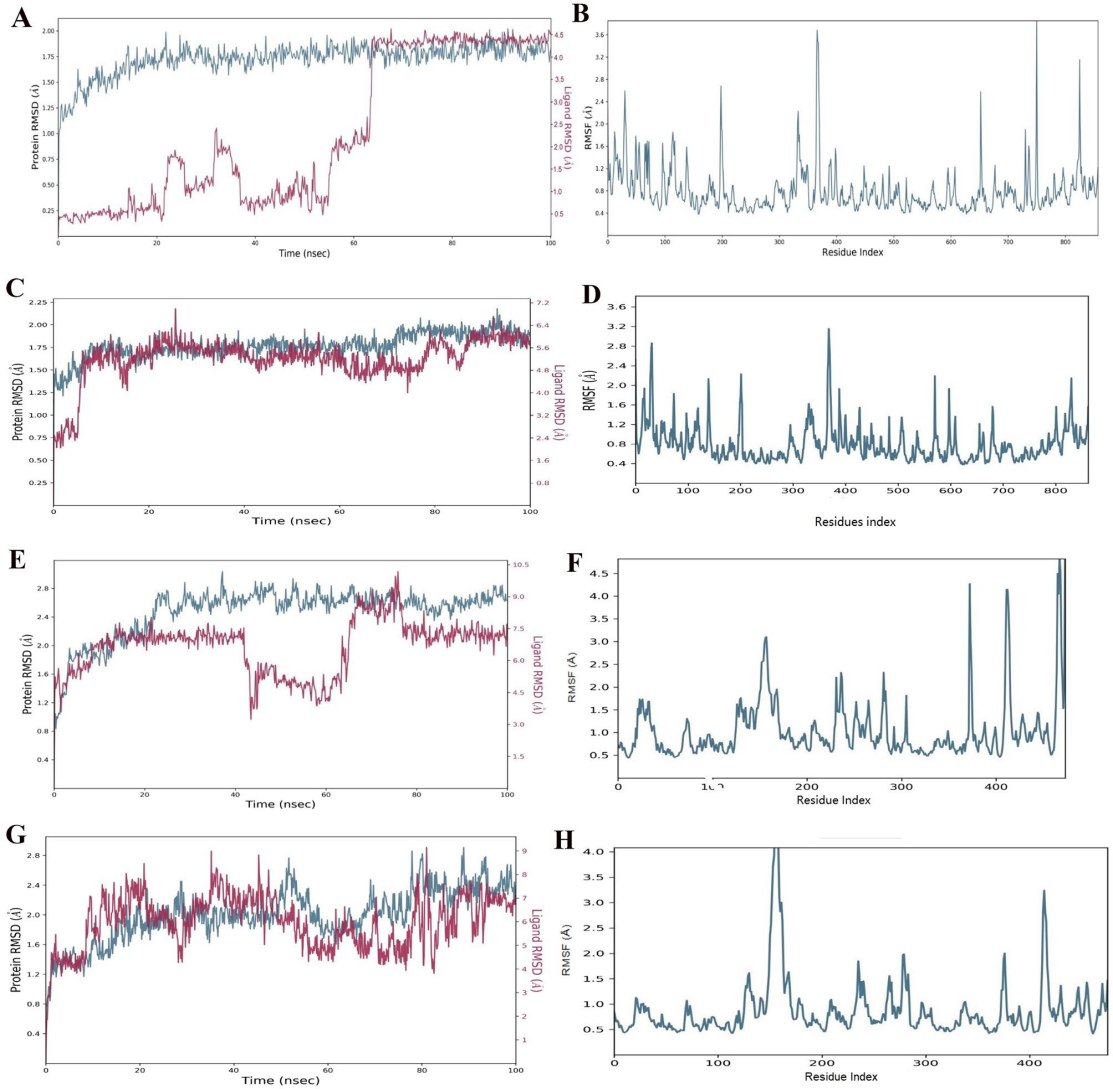
RMSD plot of α-glucosidase with ligand S1 via MD simulation at 100 ns (**A**), RMSF plot of α-glucosidase protein (**B**), MSD plot of α-glucosidase with ligand Acarbose via MD simulation at 100 ns (**C**), RMSF plot of α-glucosidase protein (**D**); RMSD plot of α-amylase with ligand S1 via MD simulation at 100 ns (**E**), RMSF plot of α-amylase protein (**F**); RMSD plot of α-amylase with ligand acarbose via MD simulation at 100 ns (**G**), RMSF plot of α-amylase protein (**H**).

A root mean square deviation (RMSD) plot was employed to find the stability of ligand molecule with protein during the simulation run at 100 ns. The stability of the protein (2QMJ) complex with ligand S1 can be seen after 60 ns in the RMSD plot and little instability from 0 to 60 ns that ranges between 0.25 to 1.75 Å (Fig. 2A). During the simulation run of 100 ns, acarbose also exhibits stability with slight fluctuation between 0 to 5 ns ranging from 0.75 to 1.25 Å (Fig. 2C). While complex of Ligand S1 with protein (7TAA) showed stability from 0 to 20 ns and 65 to 75 ns and depicted fluctuation between 20 to 65 ns and 75 to 100 ns that ranges between 2.3 to 2.5 Å (Fig. 2E). An amylase complex with acarbose positive control exhibits good stability during the simulation run and no visible fluctuation in the overall run. (Fig. 2G).

#### RMSF analysis

A stable and satisfactory RMSF fluctuation can be seen by a complex of α-glucosidase protein (2QMJ) and S1 ligand during a simulation run with a ligand contact maximum at 3.6 Å (Fig. 2B). RMSF analysis performed on already bounded ligand acarbose with α-glucosidase (2QMJ) shows satisfactory and stable RMSF fluctuations with the highest ligand contact at 3.2 Å (Fig. 2D). Stable fluctuation during the simulation run can be observed by α-amylase (7TAA) with maximum ligand contact at 5 Å and 4 Å, respectively (Fig. 2F,H).

### Enzyme inhibition analysis

To check the inhibitory potential of synthetic compounds for α-glucosidase and α-amylase enzymes, enzyme inhibition analysis was employed. The microdilution method was applied for MIC analysis to determine the IC50. Compound S1 shows good % inhibition and less IC50 as compared to control acarbose. The α-glucosidase enzyme compound S1 has 72% inhibition with IC50 3.91 µM, which is less than the standard drug (acarbose IC50 58.8 µM). The α-amylase S1 has 46% inhibition and 8.89 µM IC50, while acarbose shows low % inhibition (33%) and high IC50 (28.8 µM) as compared to S1 (Table 4).

**Table 4:** % inhibition and IC50 against α amylase and α-glucosidase.

Compound	α-glucosidase		α-amylase	
	% inhibition	IC50 (µM)	% inhibition	IC50 (µM)
S1	55 ± 0.047	3.91	46 ± 0.091	8.89
Acarbose	37 ± 0.026	58.8	33 ± 0.042	28.5

### Enzyme kinetic study

**Figure 3:**
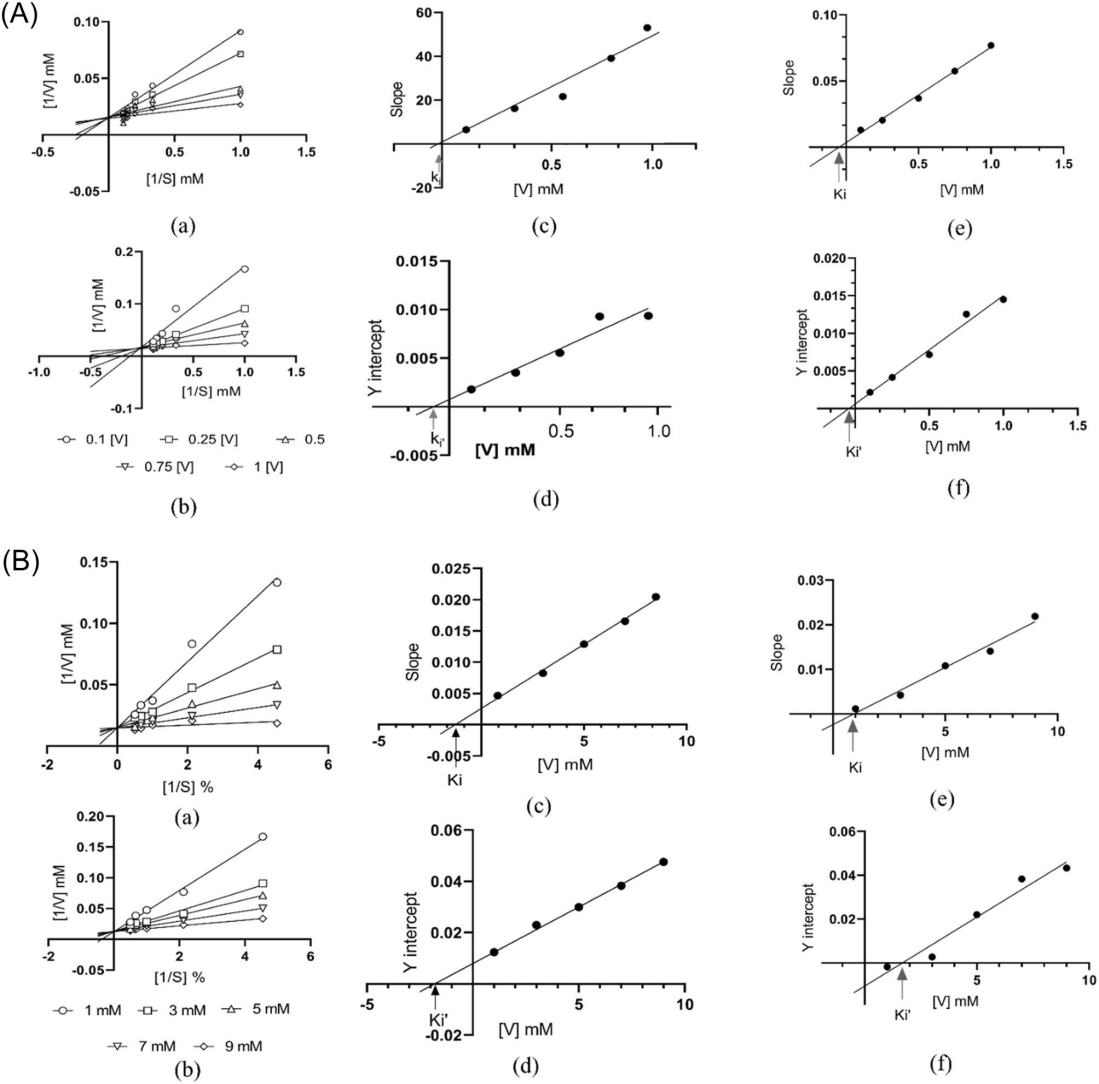
Kinetic study of α-glucosidase, data collected through in-vitro experiment was further analyze via GraphPad prism (**A**). (**a**)-Lineweaver–Burk plot of acarbose, (**b**)-Lineweaver–Burk plot for Compound S1, (**c**–**e**)-Assessment of Ki constant through slope versus inhibitor plot (S1 and acarbose respectively), (**d**–**f**)-Y of S1 and acarbose for the Assessment of Kiʹ constant through -intercept versus inhibitor plot (S1 and acarbose respectively). Kinetic study of α-amylase (**B**). (**a**)-Lineweaver–Burk plot of acarbose, (**b**)-Lineweaver–Burk plot for Compound S1, (**c**–**e**)-Assessment of Ki constant through slope versus inhibitor plot (S1 and acarbose respectively), (**d**–**f**)-Y of S1 and acarbose for the Assessment of Kiʹ constant through -intercept versus inhibitor plot (S1 and acarbose respectively).

**Figure 4:**
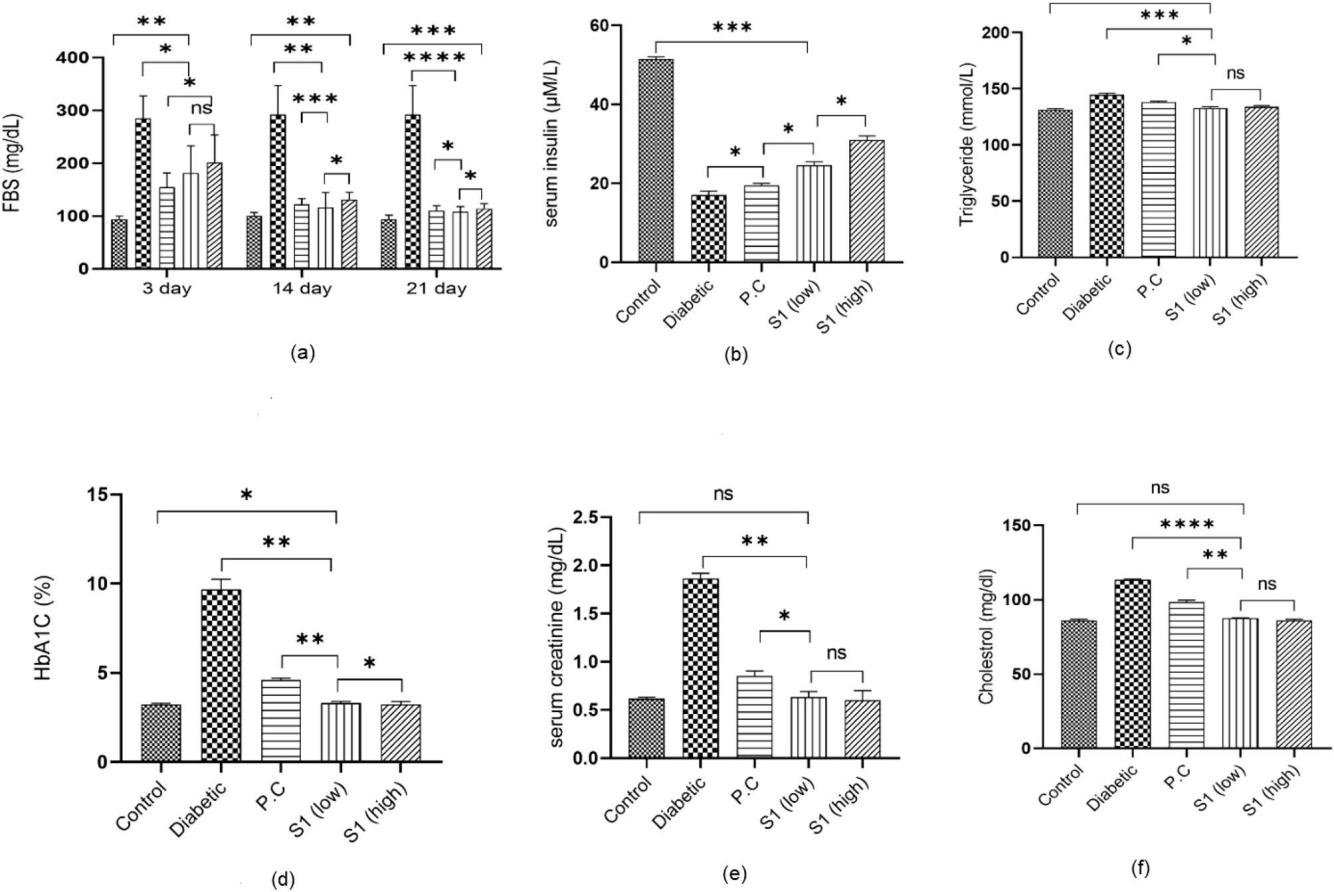
Biochemical investigation (Compound S1) of mouse blood performed through different kits. (**a**) FBS level of mice, (**b**) Distribution of serum insulin, (**c**) Distribution of triglyceride, (**d**) Hba1c, (**e**) Distribution of serum creatinine, (**f**) Distribution of cholesterol level. (*p* > 0.5 = not-significant (ns), ‘*’ = *p* < 0.05, ‘**’ = *p* < 0.01,’ ***’ = *p* < 0.001, ‘****’ = *p* < 0.0001.

Inverse substrate concentration was plotted at the x-axis and the inverse of inhibitor concentration was put on the y-axis for the investigation of the Lineweaver–Burk plot. The resulting graph for all the concentrations gives a straight line that intercepts at a point in the first quadrant indicating a competitive type of inhibition. Secondary graphs were plotted to demonstrate the inhibition constant and dissociation constant ki and ki respectively. The possibility of inhibitor on an allosteric binding site can be indicated through the value of ki, strong binding of inhibitor with enzyme is inversely proportional to the value of ki constant. A low value of ki (− 0.186 µM) and ki′ (− 0.691 µM) was exhibited by compound S1, which indicates that S1 inhibits the enzyme competitively (Fig. 3). It is an indication of solid binding within ligands and proteins. The same competitive inhibition can be observed in acarbose (Fig. 3). Ki (− 1.267 µM) and Ki′ (− 1.78 µM) were observed for amylase inhibition by compound S1 with competitive inhibition (Fig. 4).

### In vivo study

#### Biochemical investigation

Compound S1 was checked in a mouse model to illustrate the effects of compounds within a living body. After induction of diabetes in healthy mice, they were checked for fasting blood sugar level, serum insulin, serum creatinine, triglyceride, and HbA1c level. A graphical representation of biochemical analysis is shown in Fig. 4. Fasting glucose level was measured after 3, 14, and 21 days of the treatment. The Control group has a glucose level near 100 mg/dL in fasting. The diabetic control group showed > 300 mg/dL of sugar in the blood but the treatment group for S1 displayed a significant decrease in sugar levels with single and double doses (110 mg/dL,). On day 21, diabetic and treatment groups showed major differences and the data is more significant. Creatine phosphate comes from muscles and protein catabolism converts it into creatinine, a waste product for the body. The body removes this creatinine from the body through urine. However, when the kidney has impaired function, it becomes unable to filter the blood, resulting in high creatinine levels within the blood. The diabetic control group shows a high creatinine level (1.9 mg/dL). In comparison, the treated group shows nearly a normal amount of creatinine (0.6 mg/dL) in blood compared to the acarbose-treated group (0.8 mg/dL). The diabetic control and treatment groups have significant data with a p-value of 0.0028. At the same time, no major difference is seen between the control and treatment groups as the p-value is not significant. Serum insulin level is released by pancreatic beta cells. Normal mice have serum insulin levels of about 15 µM/L. However, treated group S1 shows a measurable increase in serum insulin level that is 25 µM/L with a single dose of S1 and 30 µM/L with a double dose of S1. HbA1C level in blood proves the average glucose level of the past 3 to 5 months. This test % of the glycated haemoglobin molecule is measured and shows the high blood sugar level within the blood from the past 3–5 months. The diabetic control group shows a high level of glycated haemoglobin 9% while the treatment group has only 3% of glycated haemoglobin compared to the acarbose control group (5%). Hepatic cells produce cholesterol and the body also gets cholesterol from food. High cholesterol levels indicate the accumulation of fats into blood arteries leading to heart failure due to high blood pressure and blockage of arteries. The diabetic untreated mice group has an increased level of cholesterol treated group of S1 illustrates low cholesterol (85 mg/dL) as compared to the diabetic (120 mg/dL) and acarbose treated control group (100 mg/dL) within the blood. Triglyceride is a fat, and its level should be low in the blood. High glyceride levels increase the risk of heart disease and metabolic syndrome. Mice treated with S1 Compound shows low triglyceride level (0.6 mg/dL) as compared to diabetic (1.9 mg/dL), and positive control group (0.8 mg/dL) (Fig. 4).

#### Histology analysis

**Figure 5:**
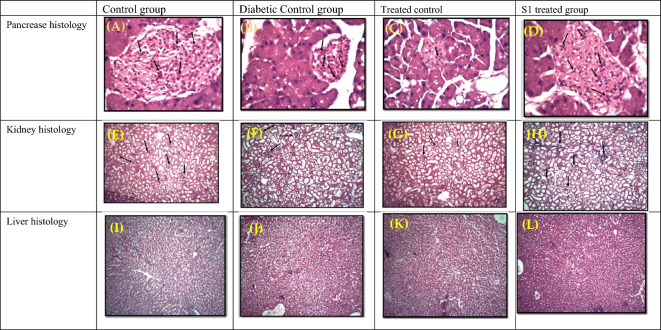
Mice were dissected and desired organs were separated and immediately kept in cold PBS solution, after that histology was performed and examination of sample was made through compound microscope. Pancreas Histology (**A**) Control group, (**B**) Diabetic control group, (**C**) Treated control (acarbose), (**D**) S1 treated group. Kidney histology. (**E**) Control group, (**F**) Diabetic control group, (**G**) Treated control (acarbose), (**H**) S1 treated group. Liver histology. (**I**) Control group, (**J**) Diabetic control group, (**K**) Treated control (acarbose), (**L**) S1 treated group.

Mice were dissected after 21 days of treatment and the kidney, liver, and pancreas were separated to check the drug effects on these organs. The pancreas contains islets of Langerhans pancreas contain three different types of cells (alpha, beta and delta) that are involved in the production of three hormones (glucagon, insulin and somatostatin) with distinct functions. Pancreatic beta cells produce insulin. Alloxan’s main target is the destruction of beta cells which reduces insulin production and increases the glucose level in the blood. The control group (non-diabetic) has well-shaped beta cells, while the diabetic group exhibited less volume of pancreatic islets, indicating the cells' necrosis with decreased volume of pancreatic islets and bets cells (Fig. 5). The glomerulus is a region in the kidney that filters the blood and tubules collect the unabsorbed water and waste and remove it from the body. Any administered kidney drug orally must filter that leaves an impact on the nephron of kidney cells and necrosis of cells to start diminishing the filtration activity of the glomerulus. The diabetic mice group (B) shows necrosis of cells, and the treated group with compound S1 (D) demonstrates less necrosis and more volume of cells indicating the drug has improved the insulin secretion (Fig. 5). The liver is involved in regulating the metabolic activities of the body detoxifying the toxic chemicals and metabolizing drugs. Any reaction due to a toxic drug leads to the necrosis of hepatic cells and affects the body's metabolic activities**.** In the control group (A) hepatic cells are densely packed and have fewer spaces than in the diabetic control group (B). While treatment group (D) also shows less necrosis and hepatic cells are tightly packed (Fig. 5). Overall S1 synthetic compound proved less toxic in the kidney, liver, and pancreas. It increases insulin secretion by pancreatic beta cells, lowering waste product accumulation within the body due to prolonged hyperglycemia.

### Proposed mode of action of pyrazolobenzothiazine derivative (S1)

**Figure 6:**
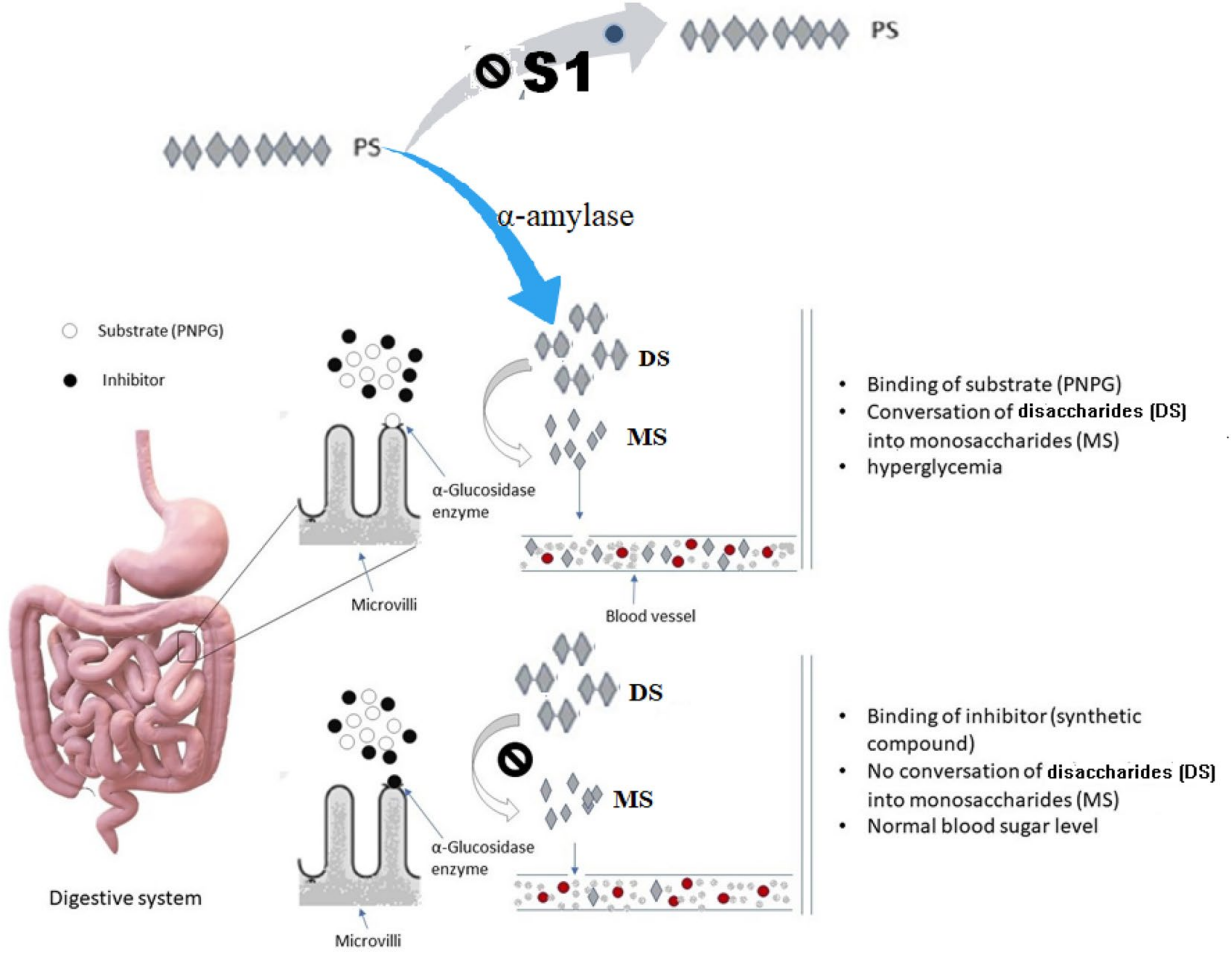
Schematic representation illustrating the potential mode of action for the proposed inhibitor S1.

The proposed synthetic compound S1 demonstrated significant inhibition of both α-glucosidase and α-amylase enzymes in vitro, demonstrating its ability to block the breakdown of complex carbohydrates such as starch into glucose (Fig. 6). It is proposed that the ability of salivary/pancreatic α-amylases to break the α-(1 → 4)-D-glycosidic bonds of polysaccharides converting them into oligosaccharides or disaccharides such as maltose is hampered by S1. On the other hand, S1 blocks α-glucosidase in the micro villi of intestine and consequently disaccharides are not converted into glucose as shown in Fig. 6. Moreover, kinetic studies also confirmed that S1 competitively prevented both α-glucosidase and α-amylase, indicating it competes with substrates.

## Discussion

Diabetes mellitus is a metabolic disorder in which pancreatic beta cells don’t produce enough insulin that can lower hyperglycemia in the blood. As cells become unable to metabolize sugar, their level increases in the blood. Another reason for increased sugar levels is that the body’s cells become resistant to the use of insulin. Uncontrolled diabetes mellitus causes different complications. Loss of vision, cardiovascular diseases, and renal impairment due to damage to nerves and blood vessels are due to long-term diabetes. In 2012, about one and a half million people died due to diabetes and its complications. In this study, in silico, in vitro, and in vivo identification was carried out to check the antidiabetic potential of pyrazolobenzothiazine compound against α-glucosidase and α-amylase. Synthetic compounds were docked with α-glucosidase and α-amylase to discover their interaction as enzyme inhibitors. Enzyme inhibition assay and molecular docking of synthetic compounds are reported, indicating the antidiabetic potential of synthetic compounds with the minimum dose and less toxicity and gastrointestinal discomfort^24,25^. Molecular docking is the best way to find virtual hits. Different studies reported molecular docking studies of synthetic compounds. Compound S1 has best-fit values compared to already reported studies^26,27^. A compound that showed the highest binding affinity and higher number of interacting residues was selected for ADMET and MD simulation analysis.

Based on ADMET and drug likeliness, compound S1 was evaluated. ADMET profiling of potential compound S1 shows no side effects on the compound's absorption. ADMET-associated properties of compounds for various + models such as P-glycoprotein substrates, BBB penetration, and gastrointestinal absorption indicate potent values that strongly prove the synthetic drug is a promising candidate. S1 also passed different drug-like rules, including Lipinski’s rule of five, Veber rule, Egan rule, and GSK 4/400 rule with non-carcinogen ability. The compound has a molecular weight < 400 Dalton with a logp value < 4 and no more than 10 hydrogen bond acceptors and donors are present. ADMET analysis of synthetic compounds showed poor drug-like properties compared to compound S1^28^. These parameters showed that compound S1 (pyrazolobenzothiazine) has strong drug-like properties and ADMET profiling^29^. Compound S1 also showed stability with α-glucosidase after 60 ns ranges between 0.25 to 1.75 Å and α-amylase S1 showed stability from 0 to 20 ns and 65 to 75 ns ranges between 2.3 to 2.5 Å with acceptable RMSD value and showed maximum hydrogen bonding interaction between the protein–ligand complex that indicates the stability of the ligand–protein complex (Fig. 2A–F). Results proved the potential and significant stability of S1 with α-glucosidase and α-amylase residues. Synthetic drugs reported in the different articles showed stable conformation with enzymes during the MD simulation run^30,31^. Likewise, compound S1 also exhibit stable conformation throughout the simulation run of 100 ns.

After in silico analysis, S1 was evaluated for α-glucosidase and α-amylase inhibition. Enzyme inhibition analysis proved that compound S1 with less IC50 value blocks the catalytic site of the enzyme compared to standard drug acarbose (Table 4). Diarrhoea, flatulence, and toxicity are some side effects reported with acarbose. However, when synthetic compound S1 was administered, no significant side effect was observed in the mice model. Many inhibitors are registered and used as a drug in the market with high IC50 values for α-glucosidase enzyme^32,33^. Acarbose has also been reported^34,35^. Compound S1 is a safe and conventional alternative for the inhibition of α-glucosidase and α-amylase. Anti-depressant, anti-microbial and anti-oxidant activities of pyrazolobenzothiazine derivatives are already reported^36,37^. Compound S1 exhibits a low value of IC50 for α-glucosidase as compared to the already reported synthetic inhibitor that showed IC50 > 50 µM^35^. As S1 has 3.91 µM IC50 it proves more efficient than the inhibitor reported in the article^38^. The other researcher proves validation of synthetic compound as a potent antidiabetic inhibitor through molecular docking and in vitro biochemical analysis of synthetic compound^20,27,35^.

Furthermore, the graph plotted for enzyme kinetics divulged that compound S1 inhibits the enzyme's catalytic activity competitively with a low value of Ki and Ki′ (Table 3). In non-competitive inhibition, the inhibitor may bind to another site rather than the binding site and cause a conformational change in enzyme structure. On the other hand, the inhibitor binds to a specific active site and seizes the enzyme's catalytic property without changing the enzyme structure in the competitive type of inhibition.

While in competitive inhibition, the inhibitor binds at the active site and seizes the substrate's binding without changing the enzyme structure. Secondly, in non-competitive inhibition, the substrate remains bound; with the enzyme because the inhibitor does not bind at the active site, and the site is available for the binding of the substrate. Grabbing the catalytic property of the enzyme’s competitive inhibition is an excellent way. In a competitive type of inhibition, the binding site is not free; for the binding of substrate. The compound S1, with both enzymes, has shown the same sort of inhibition. Kinetic study of synthetic compounds reported in different studies indicates the competitive type of inhibition^26,39,40^, and some synthetic compounds showed non-competitive inhibition^41^.

Biochemical analysis conducted on the blood of mice administered with the drug showed drastic changes in blood sugar levels compared to the conventional drug and diabetic control groups. Creatinine levels were also low in the blood of mice provided with the drug, which revealed normal renal functioning. It shows that this drug has no negative effects on kidneys or their functioning. Moreover, insulin levels were higher in mice provided with the drug compared to positive and diabetic groups. This drug has more inhibiting strength and doesn’t show negative effects on renal functioning and higher insulin levels, which is mandatory for controlling diabetic complications and reducing symptoms. The biochemical analysis also revealed low levels of Hb1Ac and cholesterol, which vouched for low levels of blood sugar. The assessment of synthetic compounds as antidiabetic agents in vivo trials^42–44^ showed lower glucose and normal insulin levels and creatinine with good ADMET profiling. Compound S1 also displayed no toxicity within the liver and pancreatic tissues, and the glomerulus region of the mouse’s kidney had less necrosis than the diabetic control group. Synthetic compounds are majorly non-toxic and don’t damage the body’s tissues^45^; however, few reported synthetic compounds showed high necrosis in the liver and kidney^46^. The molecular docking and simulation analysis revealed that test compound S1 demonstrated the most favourable binding pose. Moreover, it exhibited a lower IC50 value and significantly reduced necrosis. Additionally, it demonstrated enhanced control over complications related to diabetes. Our in-silico study played a crucial role in identifying the optimal binding pose within the complex, which was subsequently subjected to further evaluation through both in vitro and in vivo analyses. The described parameters serve as robust predictors for identifying potential drug candidates, and the proposed drug demonstrates superior fit values when compared to acarbose and other inhibitors studied previously. Compound S1 stands out in in vitro studies, displaying the optimal binding pose and fit values, surpassing the efficacy of acarbose and other drugs in the comparison. In the in vivo study conducted on a diabetic mouse model, it becomes evident that compound S1 emerges as the most promising inhibitor for the targeted enzyme, alpha-glucosidase. The findings suggest that S1 has the potential to effectively mitigate diabetic complications, showcasing its superiority over other compounds in this regard^47^.

Low toxicity and side effects were observed within the living body after administration of the synthetic compound^48^. To commercialize the S1 compound as a drug, more validation like cell toxicity analysis and human trials are needed. Compound S1 proved more potent than the conventional drug in minimizing diabetic complications Test sample exhibited no side effects, and it is cheaper than the standard drug acarbose^47^. In addition, in diabetic patients, acarbose cannot be produced to improve the functionality of beta cells and insulin secretion^49^. As reported in this study, the S1 compound assists the beta cell’s condition with maximum insulin secretion and a lower rate of diabetic-related diseases (nephropathy, liver toxicity).

## Conclusions

In this research work, we used S1 inhibitor to analyse its efficacy in reducing activity of α -glucosidase and α-amylase by molecular docking and animal studies on mice. This S1 compound demonstrated significantly better inhibitory efficiency with low IC50 values as compared to standard reference (α-glucosidase: 3.91 µM vs 58.8 µM; α-amylase: 8.89 µM vs 28.5 µM) and maximum inhibition percentages of (α-glucosidase: 55% vs 37%; α-amylase: 46% vs 28.5%). In vivo studies conducted on mice models showed outstanding biochemical characteristics, including reduced blood glucose levels (110 mg/dL), maximum insulin secretion (30 µM/L), controlled cholesterol (85 mg/dL), triglyceride (0.6 mg/dL), and creatinine (0.6 mg/dL), together with minimum glycosylated hemoglobin of 3%. Notably, the in vivo models exhibited minimum rate of necrosis and toxicity in vital organs such as the liver, kidney, and pancreas. Furthermore, molecular dynamics (MD) simulation analysis highlights the stability of the synthetic compound S1 with minimum conformational changes, maximum interacting residues, low root-mean-square deviation (RMSD), and promising drug-like possessions. Finally, synthetic compound S1 demonstrated huge potential in managing hyperglycemia and diabetic-related complications by showcasing powerful inhibitory action against key enzymes related to glucose metabolism. However, further comprehensive studies are imperative to thoroughly evaluate the compound's toxicity profile before its clinical translation.

## Data Availability

All the data are presented in this manuscript.

## Abbreviations

MOE: Molecular operating environment
ADMET: Absorption, distribution, metabolism, excretion, toxicity analysis
PNPG: *p*-nitrophenyl-α-D-glucopyranoside
PDB: Protein Data Bank
Ki: Inhibitory constant
Ki': Dissociation constant
